# Determinants of long-term survival in late HIV presenters: The prospective PISCIS cohort study

**DOI:** 10.1016/j.eclinm.2022.101600

**Published:** 2022-08-03

**Authors:** Raquel Martin-Iguacel, Juliana Reyes-Urueña, Andreu Bruguera, Jordi Aceitón, Yesika Díaz, Sergio Moreno-Fornés, Pere Domingo, Joaquín Burgos-Cibrian, Juan Manuel Tiraboschi, Isik Somuncu Johansen, Hortensia Álvarez, Josep M Miró, Jordi Casabona, Josep M Llibre, PISCIS study group

**Affiliations:** a Centre of Epidemiological Studies of HIV/AIDS and STI of Catalonia (CEEISCAT), Health Department, Generalitat de Catalunya, Badalona, Spain; b Department of Infectious Diseases, Odense University Hospital, Odense, Denmark; c Infectious Diseases Unit, Hospital Universitari de la Santa Creu i Sant Pau, Barcelona, Spain; d Department of Infectious Diseases, Hospital Universitari de la Vall d'Hebron, Barcelona, Spain; e Department of Internal Medicine and Infectious Diseases, Hospital Universitari de Bellvitge, Hospitalet de Llobregat, Barcelona, Spain; f Infectious Diseases Unit, University Hospital of Ferrol, Spain; g Hospital Clínic-Institut d'Investigacions Biomèdiques August Pi i Sunyer, University of Barcelona, Barcelona, Spain; h CIBERINFEC, Instituto de Salud Carlos III, Madrid, Spain; i Department of Paediatrics, Obstetrics and Gynecology and Preventive Medicine, Universitat Autònoma de Barcelona, Badalona, Spain; j Fundació Institut D'investigació En Ciències De La Salut Germans Trias I Pujol (IGTP), Badalona, Spain; k Spanish Consortium for Research on Epidemiology and Public Health (CIBERESP), Instituto de Salud Carlos III, Madrid, Spain; l Infectious Diseases Department and Fight Infections Foundation, University Hospital Germans Trias i Pujol, Badalona, Barcelona, Spain

**Keywords:** HIV, Late presenters, Delayed HIV diagnosis, Immune recovery, Immune response, Integrase inhibitors, Mortality

## Abstract

**Background:**

Late HIV diagnosis (i.e CD4≤350 cells/µL) is associated with poorer outcomes. However, determinants of long-term mortality and factors influencing immune recovery within the first years after antiretroviral treatment (ART) initiation are poorly defined.

**Methods:**

From PISCIS cohort, we included all HIV-positive adults, two-year survivors after initiating ART between 2005–2019. The primary outcome was all-cause mortality according to the two-year CD4 count. We used Poisson regression. The secondary outcome was incomplete immune recovery (i.e., two-year CD4<500 cells/µL). We used logistic regression and propensity score matching.

**Findings:**

We included 2,719 participants (16593·1 person-years): 1441 (53%) late presenters (LP) and 1278 non-LP (1145 non-LP with two-year CD4 count >500 cells/µL, reference population). Overall, 113 patients (4·2%) died. Mortality was higher among LP with two-year CD4 count 200–500 cells/µL (aMRR 1·95[95%CI:1·06-3·61]) or <200 cells/µL (aMRR 4·59[2·25-9·37]).

Conversely, no differences were observed in participants with two-year CD4 counts >500 cells/µL, regardless of being initially LP or non-LP (aMRR 1·05[0·50-2·21]). Mortality rates within each two-year CD4 strata were not affected by the initial CD4 count at ART initiation (test-interaction, *p* = 0·48). The stronger factor influencing immune recovery was the CD4 count at ART initiation. First-line integrase-inhibitor-(INSTI)-based regimens were associated with reduced mortality compared to other regimens (aMRR 0·54[0·31-0·93]) and reduced risk of incomplete immune recovery in LP (aOR 0·70[0·52-0·95]).

**Interpretation:**

Two-year immune recovery is a good early predictor of long-term mortality in LP after surviving the first high-risk 2 years. Nearly half experienced a favorable immune recovery with a life expectancy similar to non-LP. INSTI-based regimens were associated with higher rates of successful immune recovery and better survival compared to non-INSTI regimens.

**Funding:**

Southern-Denmark University, Danish AIDS-foundation, and Region of Southern Denmark.

**Research in context**

**Evidence before this study**

Three cohort studies and two randomized controlled trials (RCT) have indicated that integrase strand transfer inhibitors (INSTI)-based regimens might offer a benefit in immune recovery compared to other ART regimens. However, these cohort studies included both late and non-late presenters, and the last is under-represented in RCT. Knowledge on determinants of long-term mortality and immune recovery in late presenters is scant.

**Added value of this study**

Our results provide new evidence that ART initiation with an INSTI-based regimen is associated with a more favorable immune response in late presenters and with improved long-term survival especially compared to protease inhibitors.

Besides, PLWH surviving the first two years after ART initiation and reaching a CD4 cell count threshold above 500 cells/μL achieved similar survival rates to non-late presenters, irrespective of their baseline CD4 count at ART initiation. Thus, ART-associated immune recovery at two years was a good early predictor of long-term mortality.

**Implications of all the available evidence**

The long-term survival prognosis for late presenters can be assessed upon immune recovery seen two years after ART initiation. Those achieving an early favorable response improve their life expectancy to the same level as non-late presenters, thus narrowing the gap between late and non-late presenters. INSTI-based ART is associated with a more favorable immune response and significantly improved survival, supporting the growing body of evidence in clinical trials. Based on all knowledge available, health care programs should consider using two-year CD4 cell counts after ART initiation as an early prognostic factor for long-term survival in late presenters. Also, INSTI-based regimens should be considered the preferred treatment choice, particularly in late HIV presenters.

Alt-text: Unlabelled box

## Introduction

HIV-associated morbidity and mortality have decreased dramatically since the introduction of antiretroviral therapy (ART) in 1996. Today, people living with HIV (PLWH) timely diagnosed and starting treatment with CD4 counts ≥350 cells/μL have life expectancies close to the general population.^1^, ^2^, ^3^, ^4^, ^5^, ^6^, ^7^, ^8^ Still, despite the enormous advances achieved in the field, approximately half of PLWH in Western countries are diagnosed at a late disease stage, defined as CD4 cell count below 350 cells/μL or presentation with an AIDS-defining event, regardless of the CD4 cell count.^9^

This late HIV diagnosis significantly reduces life expectancy while increasing the frequency of HIV-related comorbidities,^10^, ^11^, ^12^, ^13^, ^14^ it is associated with higher health care costs,^15^ and onward transmission in the population.^16^^,^^17^ Furthermore, 10–29% of PLWH presenting late to care fail to achieve a satisfactory immune recovery despite virologically effective ART. These patients, categorized as “immunological non-responders”, have higher mortality and a higher risk for AIDS- and non-AIDS-related morbidity.^12^^,^^18^^,^^19^

Various observational studies of late presenters (LP) have reported high mortality rates within the first year after ART initiation, mainly due to advanced AIDS-defining conditions present at that time, which tends to decrease after this period.^20^^,^^21^ Thus, CD4 counts at ART initiation, might not precisely predict long-term survival in these patients as the estimation might be distorted by this initial high-mortality period.

This study explores the performance of an early predictor for long-term survival after ART initiation, skipping this initial high-risk time. Furthermore, the influence of the first-line ART on the clinical trajectories of LP is poorly understood. Various studies have described that first-line ART with integrase strand-transfer inhibitor (INSTI)-based regimens could be associated with an improved immune response with greater CD4 gain.^22^, ^23^, ^24^, ^25^ However, how these first-line regimens influence adequate immune recovery and long-term survival in LP has not been assessed.

In this prospective multicentre, ongoing cohort of PLWH in our area, we aimed to investigate whether CD4 cell counts 2 years after ART initiation was a good early predictor of long-term survival in LP compared with a non-LP group with an optimal two-year immune response and explored determinants of survival in these patients, including the potential impact of INSTI as first-line regimen. Furthermore, we assessed the predictors of adequate immune recovery within this period.

## Methods

### Study design and data sources

This is a multicentre, prospective, population-based longitudinal analysis conducted in Catalonia, an autonomous region located in north-easter Spain. On January 1, 2021, Catalonia had a population of 7·7 million citizens and an estimated HIV prevalence among the adult population of 0·4%.^26^ The Catalan healthcare system provides universal, tax-funded healthcare to all citizens. ART is provided free-of-charge.

Data were retrieved from the PISCIS Cohort project (Catalonian and Balearic Islands HIV cohort) and PADRIS (Public Data Analysis for Health Research and Innovation Program). For this study, only data from the Catalan PISCIS cohort were used. Briefly, PISCIS is an ongoing, prospective, multicentre, population-based cohort ongoing from 1998 that includes all PLWH aged ≥16 years followed in one of the 16 collaborating hospitals from Catalonia, representing 84% of all diagnosed PLWH.^27^ Data are updated yearly and include demographics, date of HIV diagnosis, AIDS-defining events, ART, and measurement of CD4 cell count and plasma HIV-RNA over time. PADRIS is a central research-oriented data repository which gathers, and cross matches real-world health data generated by the public health system of Catalonia (SISCAT), provided by the Catalan Agency for Health Quality and Evaluation (AQuAS).The database includes comorbidity data from hospital discharge diagnoses and primary health care from 2005 according to the International Classification of Disease 10^th^ revision (ICD-10).^28^ Mortality data was obtained cross matching the information with PISCIS and PADRIS databases and the national mortality registry.

### Ethics approval

The PISCIS cohort has been approved by the ethics committee of the coordinating centre, and patient data extraction is allowed by the 203/2015 Decree from the Catalan Health Department. PISCIS data is owned by each individual patient, and data is eliminated if requested by the hospital or by a patient. All data is pseudo-anonymized before arriving at the coordinating center, and confidentiality is guaranteed in accordance with the provisions of the Regulation (EU) 2016/679 of the European Parliament and of the Council of 27 April 2016 on the protection of natural persons regarding the processing of personal data and on the free movement of such data and the new national Organic Law 3/2018 of December 5 on Protection of Personal Data and Digital Rights.

### Study population

We screened the *PISCIS* cohort for treatment naïve PLWH ≥18 years, who initiated ART between January 1, 2005, and June 30, 2019. Of these, we included all 2-year survivors with active follow-up and available CD4 cell count at both timepoints, ART initiation and two years after treatment start.

Patients were classified according to the CD4 cell count at ART initiation into late HIV presenters (i.e., CD4 count ≤350 cells/μL) and non-LP (i.e., CD4 count >350 cells/μL). LP were further stratified according to their CD4 count at two different time points: at ART initiation (<100 cells/μL, 100–199 cells/μL, and 200–350 cells/μL) and at 2 years after ART initiation (<200 cells/μL, 200–500 cells/μL, and >500 cells/μL). Non-LP were also stratified according to their immune recovery two years after ART initiation (≤500 cells/μL and >500 cells/μL).

### Outcomes

The primary outcome was time to all-cause mortality, setting the baseline time at two years after ART initiation. We chose a two-year time-point expecting to get rid of the bias introduced in the analysis by initial mortality related to baseline conditions in this cohort of PLWH with advanced HIV infection. The outcome was assessed for the different groups of LP based on the CD4 count immune recovery two years after ART initiation; non-LP with CD4 >500 cells/μL two years after ART initiation was used as the reference population.

The secondary outcome was incomplete immune recovery two years after ART initiation, defined as CD4 cell count ≤500 cells/μL. This was assessed in a nested case-control study design.

### Statistical analysis

Continuous variables were described as the median and the interquartile range (IQR), whereas categorical variables were presented as the frequency and percentage over available data. Missing data were not imputed because the essential variables for the analysis (i.e., CD4 count available at ART initiation and 2 years after) were among the selection criteria for the study population. We used the chi-squared test, the t-test, and the Mann–Whitney U test to compare variables between late and non-LP. For polycothomous categorical variables with a statistically significant chi-square test of homogeneity, we further provided a significance test of the difference between proportions across categories of the variable between LP and non-LP.

The survival analysis considered all-cause death, loss-to-follow-up, or end of the observation period, whichever occurred first, between the 2-year time point after the date of ART initiation to June 30, 2021. The cumulative survival of PLWH in the different groups of two-year CD4 cell count was compared using the Log-rank test. We used a Poisson regression analysis to control for the following potential confounders and to explore for prognostic factors for survival: age, gender, HIV risk group (men who have sex with men (MSM), heterosexual men, women, history of injection drug use (IDU), and other/unknown), region of birth (Spain, Europe excluding Spain, Africa, America, Asia), two-year CD4 cell count recovery (<200, 200–500, and >500 cells/μL), two-year HIV viral load (>200 vs ≤ 200 copies/mL), modified Charlson comorbidity index (CCI) at two years after ART initiation, prior AIDS-defining disease at two years after ART initiation, time-updated calendar period (2005–2009, 2010–2014, 2015–2019, according to pre-INSTI-available period, INSTI-available period, and change in guidelines to immediate ART initiation,^29^ respectively), INSTI-based regimen as ART regimen initiated in the first two years, and socioeconomic level based on AQuAS composite socioeconomic indicator used to classify the primary health areas (no economic deprivation, mild economic deprivation, and moderate/severe economic deprivation).^30^ We provide mortality rate (MR) and MR ratios (MRR) and a 95% confidence interval (CI).

CD4 cell counts at ART initiation and two years after were defined as the closest value to the index date, with a time window of 12 months before and 3 months after ART initiation and 3 months before and 12 months after for the time point at two years after ART initiation. The CCI was modified according to the information available on comorbidities from the PADRIS and excluding AIDS defining events, which was assessed separately (Table S1, Supplementary data).

We conducted a sensitivity analysis using propensity score matching (1:1 nearest-neighbor matching with caliper 0·2 on each sample) for gender, age, CD4 cell count, modified CCI, and AIDS-defining event, all at ART initiation, to assess the impact of initiation of INSTI as first- line regimen on long-term mortality. The application of these probabilities to the study population created a subpopulation in which these potential confounders were more equally distributed across both groups. The same analysis was performed for the whole database population of individuals who initiated ART, regardless of survival in the first two years or availability of CD4 counts at 2 years after ART.

To identify risk factors in the group of LP associated with incomplete immune recovery 2 years after ART initiation (defined as CD4 cell count ≤500 cells/μL), we performed a logistic regression analysis with propensity score matching on age (continuous), gender, CD4 cell count at ART initiation (<100 cells/μL, 100–199 cells/μL, and 200–350 cells/μL)) and modified CCI. We assessed the following covariates at ART initiation: age, gender, CD4 cell count, INSTI as first ART regimen, modified CCI, AIDS-defining event within 90 days after ART initiation. We provide the odds ratio (OR) and a 95% CI.

All analyses were conducted using the STATA software (v.16; Stata Corp, College Station, TX, USA).

### Role of funding

This work was supported by scholarships from the University of Southern Denmark, the Danish AIDS foundation, and Public Regional Funds from the Region of Southern Denmark. The study was investigator-driven and thus independent of any pharmaceutical company. The funding sources were not involved in study design, data collection, analyses, report writing, or decision to submit the paper.

## Results

### Study participants

**Figure 1:**
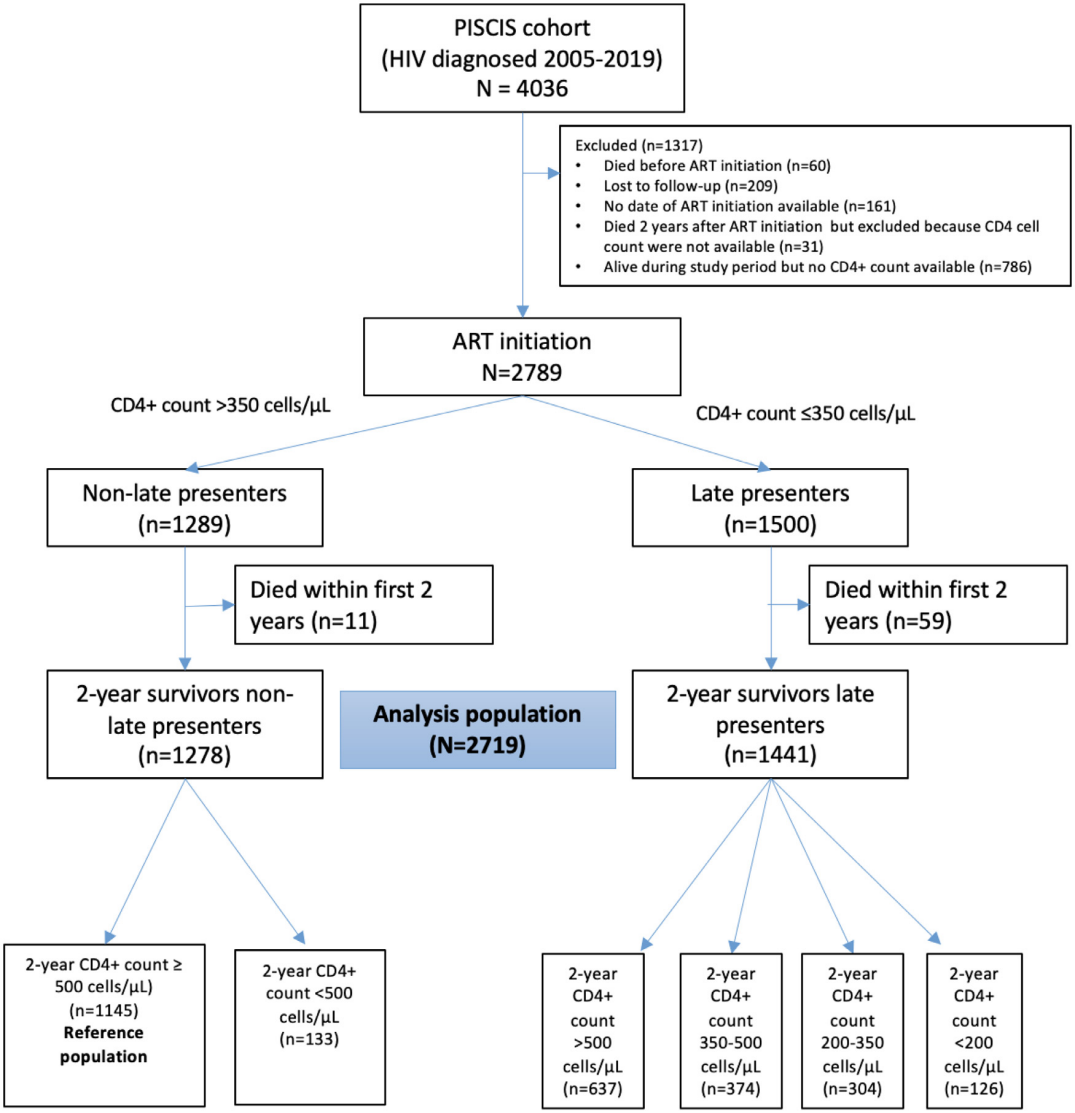
Flowchart of cohort construction.

From the PISCIS cohort, we identified 4036 PLWH starting ART between 2005 and 2019. Of them, 2719 (67·4%) were eligible for the analysis, giving rise to 16593·1 person-years of follow-up (median follow-up 5·8 years [IQR 3·2-8.9]) (Figure 1). Table S2 summarizes and compares the characteristics of included and excluded participants. Of the analysis population, 1441 (53%) were LP and 1,278 were non-LP.

Table 1 summarizes the demographic and clinical characteristics of individuals included in the analysis population, according to the defined CD4 count groups. Compared with non-LP, LP were more frequently older, heterosexual men, women, individuals with a history of IDU, of African origin, with lower educational level and higher economic deprivation. The percentage of late HIV presentation decreased in time from 77% in 2005–2009 to 41% in 2015–2020 (*p<*0·001). Among LP, 27% initiated ART with a CD4 cell count <100 cells/µL, 21·8% with 100–199 cells/µL and 51·6% with 200–350 cells/µL, and 13·2% had an AIDS-defining event at baseline. Six hundred thirty-seven (44·2%) of late presenters achieved CD4 cell counts >500 cells/µL two years after ART start. The comparator population of non-LP with two-year CD4 cell count >500 cells/µL (*n =*1145) had a median two-year CD4 count of 837 (IQR: 697-1030).

**Table 1:** Baseline characteristics.

Study population according to CD4 cell count at ART initiation				Study population according to immune recovery 2 years after ART initiation				
	Non-late presenters *n =*1278 (47·0%)	Late presenters *n =*1441 (53·0%)	*p*-value	Late presenters *n =*1441				Non-late presenters
				CD4 cell count (cells/µ)				CD4 cell count (cells/µ)
				>500 *n =*637 (44·2%)	>350-500 *n =*374 (26·0%)	≥200-350 *n =*304 (21·1%)	<200 *n =*126 (8·7%)	>500 cells/µL *n =*1145
Male, n (%)	1122 (87·8)	1202 (83·4)	0·001	549 (86·2)	314 (84·0)	238 (78·3)	101 (80·2)	1022 (89·3))
Age at ART initiation (years), median (IQR)	35 (29-42)	38 (32-45)	<0·001	36 (30-43)	39 (32-46)	41 (35-48)	40 (34-48)	35 (29-42)
Birth area, n (%)			0·015					
Spain	598 (46·8)	743 (51·6)	0·013	330 (51·8)	180 (48·1)	163 (53·6)	70 (55·6)	531 (46·4)
Europe	124 (9·7)	97 (6·3)	0·005	47 (7·4)	31 (8·3)	15 (4·9)	4 (3·2)	111 (9·7)
Africa	43 (3·4)	72 (5·0)	0·035	18 (2·8)	20 (5·4)	22 (7·2)	12 (9·5)	36 (3·1)
America	249 (19·5)	289 (20·1)	0·71	141 (22·1)	81 (21·7)	49 (16·1)	18 (14·3)	221 (19·3)
Asia	14 (1·1)	15 (1·0)	0·89	10 (1·6)	3 (0·8)	0	2 (1·6)	14 (1·2)
Unknown	250 (19·6)	225 (15·6)	0·007	91 (14·3)	59 (15·8)	55 (18·1)	20 (15·9)	232 (20·3)
HIV risk group, n (%)			<0·001					
MSM	910 (71·2)	742 (51·5)	<0·001	415 (65·2)	198 (52·9)	94 (30·9)	35 (27·8)	842 (73·5)
Heterosexual men	127 (9·9)	266 (18·5)	<0·001	85 (13·3)	65 (17·4)	85 (28·0)	31 (24·6)	109 (9·5)
Women	128 (10·0)	195 (13·5)	0·005	74 (11·6)	51 (13·6)	54 (17·8)	16 (12·7)	103 (9·0)
IDU	56 (4·4)	131 (9·1)	<0·001	26 (4·1)	38 (10·2)	36 (11·8)	31 (24·6)	44 (3·8)
Unknown	57 (4·5)	107 (7·4)	0·001	37 (5·8)	22 (5·9)	35 (11·5)	13 (10·3)	47 (4·1))
ART- regimen in the first 2 years, n (%)								
NNRTI-based regimen	570 (44·6)	723 (50·2)	0·004	338 (53·1)	197 (52·7)	131 (43·1)	57 (45·2)	508 (44·4)
PI-based regimen	350 (27·4)	629 (43·7)	<0·001	240 (37·7)	164 (43·9)	147 (48·4)	78 (61·9)	300 (26·2)
INSTI-based regimen	544 (42·6)	435 (30·2)	<0·001	198 (31·1)	109 (29·1)	83 (27·3)	45 (35·7)	508 (44·4)
Viral load at HIV diagnosis (log_10_ copies/mL), median (IQR)	4·8 (3·9-4·9)	4·9 (4·7-5·7)	<0·001	4·9 (4·7-5·7)	4·9 (4·0-5·7)	4·9 (4·7-5·7)	4·9 (4·7-5·8)	4·8 (3·9-4·9)
CD4 cell count at ART initiation (cells/ µL), n (%)								
<100 cells/µL	-	383 (26·6)		67 (17·5)	92 (24·0)	133 (34·7)	91 (23·8)	-
100-199 cells/µL	-	314 (21·8)		109 (34·7)	97 (30·9)	88 (28·0)	20 (6·4)	-
200-350 cells/µL	-	744 (51·6)		461 (62·0)	185 (24·9)	83 (11·2)	15 (2·0)	-
>350-500 cells/µL	613 (48·0)	-		-	-	-	-	509 (44·5)
>500 cells/µL	665 (52·0)	-			-	-	-	636 (55·6)
Viral load at ART initiation (log^10^ copies/mL), median (IQR)	4·4 (3·6-4·9)	4·9 (4·0-5·5)	<0·001	4·9 (4·3-5·3)	4·8 (3·9-5·4)	4·9 (3·9-5·7)	4·9 (4·1-5·7)	4·4 (3·7-4·9)
CD4 cell count 2 years after ART initiation (cells/µL), median (IQR)	802 (632-998)	469 (320-627)	<0·001	-	-	-	-	-
Detectable viral load 2 years after ART initiation (>200 copies/mL), n (%)	61 (4·8)	94 (6·5)	0·049	25 (3·9)	15 (4·0)	24 (7·9)	30 (23·8)	35 (3·1)
Unavailable viral load 2 years after ART initiation	113 (8·8)	81 (5·6)	0·001	34 (5·3)	21 (5·6)	18 (5·9)	8 (6·4)	109 (9·5)
Individuals with AIDS defining events at ART initiation^a^, n (%)	20 (1·6)	190 (13·2)	<0·001	49 (7·7)	47 (12·6)	56 (18·4)	38 (30·2)	14 (1·2)
Individuals with AIDS defining events in the first 2 years beyond ART initiation, n (%)	1 (0·1)	14 (1·0)	0·002	5 (0·8)	3 (0·8)	3 (1·0)	3 (2·4)	1 (0·1)
Modified Charlson comorbidity score at ART initiation, n (%)			0·080					
0	1158 (90·6)	1262 (87·6)		583 (91·5)	328 (87·7)	256 (84·2)	95 (75·4)	1041 (90·9)
1	76 (6·0)	108 (7·5)		32 (5·0)	25 (6·7)	31 (10·2)	20 (15·9)	67 (5·9)
2-3	36 (2·8)	60 (4·2)		20 (3·1)	18 (4·8)	14 (4·6)	8 (6·4)	30 (2·6)
≥4	8 (0·6)	11 (0·8)		2 (0·3)	3 (0·8)	3 (1·0)	3 (2·4)	7 (0·6)
Modified Charlson comorbidity score at 2 years after ART initiation, n (%)			<0·001					
0	1094 (85·6)	1146 (79·5)	0·012	551 (86·5)	301 (80·5)	221 (72·7)	73 (57·9)	988 (86·3)
1	107 (8·4)	158 (11·0)	0·11	52 (8·2)	39 (10·4)	42 (13·8)	25 (19·8)	90 (7·9)
2-3	62 (4·9)	106 (7·4)	0·058	29 (4·6)	26 (7·0)	32 (10·5)	19 (15·1)	53 (4·6)
≥4	15 (1·2)	31 (2·2)	0·67	5 (0·8)	8 (2·1)	9 (3·0)	9 (7·1)	14 (1·2)
Calendar time of ART initiation, n (%)			<0·001					
2005-2009	180 (23·4)	588 (76·6)	<0·001	232 (36·4)	148 (39·6)	147 (48·4)	61 (48·1)	141 (12·3)
2010-2014	651 (54·5)	543 (45·5)	0·001	264 (41·4)	145 (38·8)	94 (30·9)	40 (31·8))	586(51·2)
2015-2019	447 (59·1)	306 (41·0)	<0·001	141 (22·1)	81 (21·7)	63 (20·7)	25 (19·8)	418 (36·5)
Educational level, n (%)			<0·001					
None or primary education only	226 (39·2)	350 (60·8)	<0·001	134 (28·3)	90 (33·6)	92 (45·1)	34 (46·0)	190 (21·7)
Secondary education	396 (51·0)	380 (49·0)	0·008	187 (39·5)	106 (39·6)	64 (31·4)	23 (31·1)	361 (41·2)
University	350 (55·7)	278 (44·3)	<0·001	153 (32·3)	67 (25·0)	42 (20·6)	16 (21·6)	319 (36·4)
Unknown	8 (40·0)	12 (60·0)	0·53	0	5 (1·9)	6 (2·9)	1 (1·4)	7 (0.8)
Income, n (%)			0·010					
No economic deprivation	678 (53·1)	691 (48·0)	0·008	337 (52·9)	176 (47·1)	126 (41·5)	52 (41·3)	613 (53·5)
Mild economic deprivation	215 (16·8)	309 (21·4)	0·002	129 (20·3)	71 (19·0)	77 (25·3)	32 (25·4)	184 (16·1)
Moderate/severe economic deprivation	353 (27·6)	399 (27·7)	0·97	152 (23·9)	118 (31·6)	93 (30·6)	36 (28·6)	319 (27·9)
Unknown	32 (2·5)	42 (2·9)	0·51	19 (3·0)	9 (2·4)	8 (2·6)	6 (4·8)	29 (2·5)
Death 2 year after ART initiation, n (%)	23 (1·8)	90 (6·3)	<0·001	14 (2·2)	25 (6·7)	22 (7·2)	29 (23·0)	17 (1·5)

ART, antiretroviral therapy; INSTI, integrase strand transfer inhibitor; IQR, interquartile range (percentiles 25^th^ and 75^th^); MSM, men who have sex with men; IDU, injection drug use; NNRTI, non-nucleoside reverse transcriptase inhibitors; PI, protease inhibitor.

a (before or within 90 days from ART initiation).

### Mortality risk

**Figure 2:**
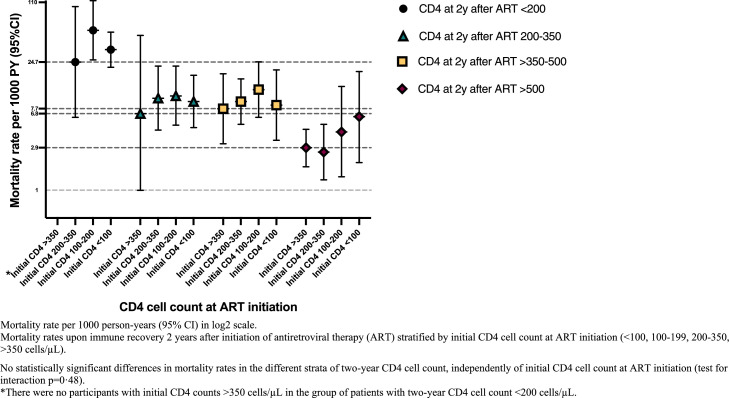
Mortality rates upon immune recovery 2 years after initiation of antiretroviral therapy stratified by nadir CD4 cell count at ART initiation.

Overall, 113 (4·2%) died during the study period (crude all-cause MR 6·8/1000 person-years; 95%CI: 5·7-8·2). Compared with non-LP with two-year CD4 count >500 cells/µL (reference population), LP with two-year CD4 count ≤500 cells/µL had higher mortality (adjusted MRR 1·95 [1·06-3·61] and 4·59 [2·25-9·37] in the groups of CD4 200–500 cells/µL and <200 cells/µL, respectively). Conversely, no significant differences were observed between the adjusted MR of non-LP and LP with two-year CD4 count >500 cells/µL (1·05 [0·50-2·21]) (Table 2 and Table S3). Within the individual strata of immune-recovery (two-year CD4 cell count >500, >350–500, 200–350, <200 cells/µL), MR were not significantly different when stratifying by initial CD4 cell count at ART initiation (>350, 200–350, 100–199, <100 cells/µL) (test for interaction *p* = 0·48) (Figure 2, Table S4). HIV viral load >200 copies/mL two years after ART initiation was also associated with increased mortality risk (1·78 [0·99-3·19]). Other factors associated with increased mortality according to the adjusted analysis included a modified CCI score at ART start ≥4 *versus* 0 and HIV risk group (i.e., heterosexual men and IDU *versus* MSM).

**Table 2:** Crude and adjusted mortality in treatment-naive individuals surviving the first 2 years after ART initiation.

ART, antiretroviral therapy; INSTI, integrase strand transfer inhibitor; MSM, men who have sex with men; IDU, injection drug use.

^a^ MRR, mortality rate ratio.

^b^ aMRR, adjusted mortality rate ratio. The multivariate analysis was adjusted for age at baseline, transmission mode, CD4 cell count recovery at 2 years after ART initiation, HIV viral load > 200 c/ml vs ≤200 c/ml at 2 years, INSTI-based regimen as first-line regimen in the first 2 years, modified Charlson comorbidity score at baseline, history of AIDS-defining event at baseline, and calendar time (time-updated).

INSTI-based regimens as first-line ART were associated with lower mortality than non-INSTI regimens (0·54 [0·31-0·93]). There was an improved survival trend when considering LP and non-LP separately, but it was not statistically significant (0·61 [0·33-1·13] and 0·25 [0·06-1·12], respectively). However, the sensitivity analysis with propensity score matching, conducted to address the potential channeling bias, did show a significant benefit of INSTI *versus* non-INSTI regimens (0·48 [0·25-0·90]) (Table S5). We did a second sensitivity analysis including all individuals starting ART regardless of survival the first two years or availability of CD4 cell count two years after ART. In line with the previous analysis, the sensitivity analysis showed that INSTI-based first-line regimens were associated with a survival benefit when compared to protease inhibitor (PI) (0·41 [0·23-0·72]) but not versus non-nucleoside reverse transcriptase inhibitors (NNRTI (0·88 [0·48-1·61]), Table S5).

**Figure 3:**
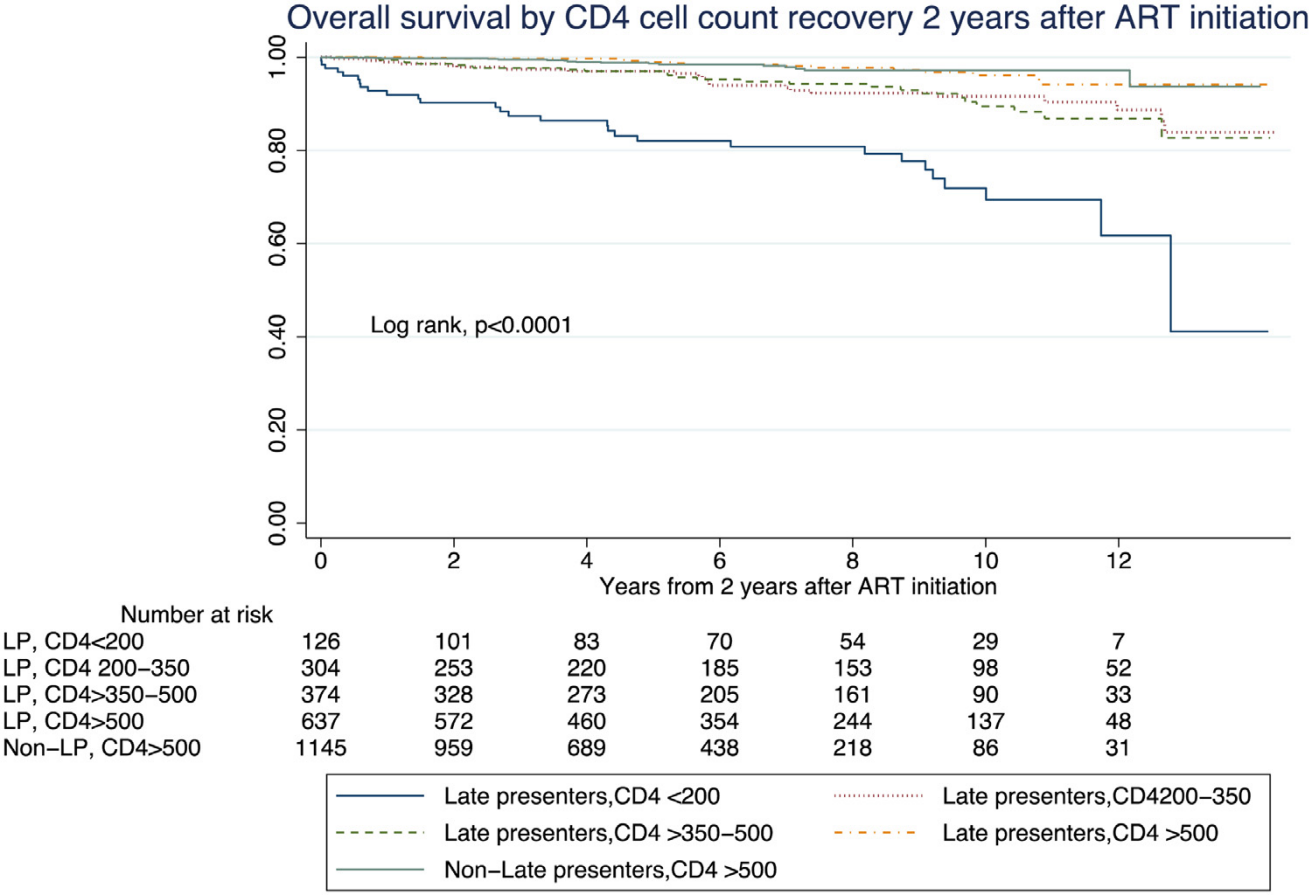
Kaplan-Meier curves for overall survival by immune recovery 2 years after antiretroviral therapy (ART) initiation (non-late presenter with CD4 counts >500 cells/µL versus late presenters with CD4 counts >500 cells/µL, late presenters with CD4 counts >350-500 cells/µL, late presenters with CD4 counts 200-350 cells/µL and late presenters with CD4 counts <200 cells/µL at 2 years after ART initiation.

The log-rank test revealed significant differences between survival curves for all-cause mortality (Figure 3). However, while LP with CD4 ≤500 cells/µL at two years had remarkably lower survival, the curve of LP with CD4 >500 overlapped with that of non-LP. As a sensitivity analysis, we excluded patients with HIV viral load >200 copies/mL or unavailable (*n =*349), with similar results (Figure S1). The long-term survival analysis comparing non-LP achieving CD4 cell count >500–699 and those with ≥700 cells/µL did not reveal significant differences between groups (0·57 [0·21-1·51]) (Tables S6 and S7).

### Risk factors for incomplete immune recovery

Table 3 summarizes the results of univariate and multivariate logistic regression analyses after propensity score matching assessing incomplete immune recovery (i.e., two-year CD4 counts ≤500 cells/µL) in LP. Lower initial CD4 counts at ART initiation strongly influenced the likelihood of incomplete immune recovery at two years, together with older age and modified CCI score 1. First-line INSTI-based therapy was associated with 30% lower odds of incomplete immune recovery in LP (adjusted OR 0·70 [0·52-0·95]). The distribution of baseline covariates in both groups of INSTI and non-INSTI before and after propensity score matching is presented in table S8. When including calendar time in the model, we found the same trend, but the results did not reach statistical significance (0·76 [0·52-1·13]). The same analysis including also non-LP with CD4 cell counts <500 cells/µL at ART initiation and using a threshold of two-year CD4 cell count ≥700 cells/µL for a satisfactory immune recovery, showed a similar benefit of first-line INSTI regimens also incorporating calendar time in the model (0·74 [0·57-0·97]) (Table S9).

**Table 3:** Variables associated with incomplete immune restoration, defined as CD4 cell count ≤500 cells/µL, 2 years after ART initiation, in individuals with CD4 cell count ≤350 cells/µL at ART initiation.

AIDS, Acquired Immune Deficiency Syndrome; ART, antiretroviral therapy; INSTI, integrase strand transfer inhibitor.

^a^ Multivariate analysis adjusted for age (categorical), gender, CD4 cell count at ART initiation, INSTI-based regimen, Charlson comorbidity score at ART initiation, and AIDS-defining event at ART initiation.

Propensity score weighting on age, gender, CD4 cell count at ART initiation, and Charlson comorbidity index at ART initiation.

## Discussion

In this population-based prospective cohort study with 2719 PLWH initiating their ART between 2005 and 2019 and surviving the first two years, 53% of them were diagnosed at a late stage (i.e., CD4 count ≤350 cells/µL). Despite this delayed ART initiation in these patients presenting late to care, 44% achieved an optimal immune recovery with a CD4 cell count >500 cells/µL at two years. LP achieving two-year CD4 cell count >500 cells/µL had comparable long-term mortality rates to non-LP, irrespective of their initial CD4 cell count. CD4 cell count recovery two years after ART initiation was a useful early predictor of long-term mortality. Exposure to an INSTI-based ART regimen in the first two years was associated with a significantly better two-year immune recovery and better long-term survival. A lower CD4 cell count, older age, and worse CCI score at ART initiation significantly increased the risk for incomplete two-year immune recovery.

Although LP have an overall increased mortality, earlier studies have shown that the risk of death and/or AIDS is highest in the first year after HIV diagnosis and decreases hereafter.^20^^,^^21^ There is less knowledge on the determinants of long-term survival after this initial high-risk period. Furthermore, many of the studies evaluating life expectancy in LP include patients initiating ART before the year 2012, thus exposed to older ART regimens.^6^^,^^31^, ^32^, ^33^, ^34^, ^35^ The available evidence seems to indicate that the association between CD4 cell count at ART initiation and mortality fades over the years as patients survive. An ART-CC cohort study of PLWH initiating ART between 1996–2001, found that there were no differences in long-term survival after 5 years among those starting ART with CD4 cell count 200–350 cells/µL or >500 cells/µL.^31^ Similarly, a Spanish cohort found that in five-year survivors without history of AIDS-defining events, there was no survival differences between those starting ART with CD4 count >350 cells/µL or with 200–350 cells/µL.^21^ A cohort study from UK including patients from 2000–2010 showed that LP improved their life span if they achieved a good immune response and suppressed HIV-1 viral load. In fact, those achieving a good early immune response, with one-year CD4 cell count ≥350 cells/μL and virological suppression, experienced a life expectancy close to the background population.^36^ However, in this study, half of the patients started ART before 2005 and no individual in the study was exposed to INSTI. Existing evidence supports that current CD4 count correlates strongly with mortality^15^^,^^33^^,^^34^ and some studies have shown that PLWH with current CD4 count ≥500 cells/μL and without history of IDU have life expectancies close to the background population.^2^ We investigated if CD4 cell count at 2-two years could be used as an early predictor of survival. Our results support that after this initial high-risk period of 2 years after HIV diagnosis in LP, likely associated with advanced AIDS-related medical conditions and complications present at diagnosis, two-year CD4 count recovery is a strong independent early predictor for long-term all-cause mortality, independently of the CD4 cell count at ART initiation. Actually, 44% of the two-year survivors successfully achieved a good immune recovery, with two-year CD4 cell counts >500 cells/µL. Importantly, the mortality rate in this subgroup of patients did not differ from that of non-LP with optimal immune response (i.e., CD4 cell counts > 500 cells/µL with a median 2-years CD4 837 [IQR: 697-1030]), thus narrowing the survival gap between late and non-LP. Only 8·7% of LP in our cohort remained with CD4 cell count <200 cells/µL at two years. These patients with immune discordance exhibited the highest mortality rates, approximately 5 times greater than non-LP. Of note, this group had a high percentage of detectable two-year viremia (24%), suggesting that increased mortality rates and immune discordance might be partially explained by ART non-adherence, aside from their low baseline CD4 count. Patients achieving CD4 cell counts between 200 and 500 cells/µL at two years still had an approximately double mortality risk compared to non-LP.

Our findings in immunological non-responders are in accordance with results from the COHERE cohort,^37^ which also found increased mortality in patients with CD4 ≤200 cells/µL compared to those above this threshold after three years of successful suppressive ART (adjusted hazard ratio 2·60 [95%CI: 1·86–3·61]). Similarly, the Italian MASTER and the Dutch ATHENA cohorts reported higher mortality risks and non-AIDS-defining events in a subpopulation of immunological non-responders.^12^^,^^18^ Participants in these studies initiated their ART in earlier calendar years (1996 to 2011 and 1998-2009, respectively), with older ART regimens. A recent published study found that in an overall cohort of PLWH initiating ART, five-year survivors with five-year CD4 <200 cells/µL remained with the worse prognosis.^32^

Several studies have described a better CD4 count recovery in patients exposed to INSTI-based regimens, mainly versus efavirenz-based regimens.^22^, ^23^, ^24^, ^25^^,^^38^, ^39^, ^40^ Initiation of ART with a INSTI-based regimen was associated with a higher CD4 count increase at three years compared to efavirenz in the SINGLE and STARTMRK phase 3 randomized studies.^23^^,^^38^^,^^39^ However, no differences were observed between Dolutegravir and Darunavir in the FLAMINGO and SYMTRI randomized trials.^41^^,^^42^ A recent collaborative cohort showed a more favourable immunologic response, defined as 25% increase in CD4 count or as reaching ≥750 cells/μL at one year, with INSTI- vs PI- or NNRTI-based regimens.^40^ This study, however, included both treatment-naïve and treatment-experienced individuals, did not assess the effect of INSTI on long-term mortality nor the specific effect on immune responses in the subpopulation of LP, and residual confounding could not be fully excluded. In our analysis, incomplete immune recovery was defined as CD4 cell count <500 cells/μL two years after ART, given that this threshold was associated with increased mortality compared with non-LP in our analysis. Choosing a higher threshold of >700 cells/μL lead to similar results both in successful immune recovery and mortality. We found 30% lower odds of incomplete immune recovery in individuals starting INSTI-based regimens, both in LP and in individuals with an initial CD4 count ≤500 cells/μL, and approximately 46% decrease in long-term mortality in the whole study population.

To our knowledge, this is the first study to identify the exposure to INSTI-based regimen first-line ART with a significantly higher probability of reaching a successful immune recovery with a significant impact on long-term mortality in LP. Nonetheless, the strongest independent risk factor for incomplete immune recovery was lower CD4 cell count at ART initiation, reinforcing the importance of timely HIV diagnosis.

Our study is strengthened by the prospective, population-based, multicentre HIV-cohort design, including 84% of all PLWH followed in Catalonia, and the long-term observation time. We had access to high-quality, real-world health data generated by the public health system providing additional information on comorbidities and mortality. We compared a fully characterized group of LP regarding their CD4 count at ART initiation and two-years after with non-LP with optimal immune response (i.e. two-year CD4 >500 cells/µL, median CD4 count 837 (IQR: 697-1030)) also including more recent calendar years and treatments. A propensity score matching helped us to obtain more comparable treatment risk groups in the cohort regarding INSTI-exposure, reducing possible channeling bias.

On the other hand, some limitations must be considered. First, the definition of LP in our study included only CD4 cell count at ART initiation ≤ 350 cells/μL and not AIDS-defining events as we focused in CD4 responses. However, few non-LP experienced an AIDS-defining event (*n =*20), so including these in the group of LP would not have significantly change the results. Second, we excluded 817 individuals from the analysis because initial (*n =*223) or two-year CD4 counts (*n =*708) were not available. Of the last group, 41% (*n =*281) corresponded to individuals whose two-year blood samples had to be collected during the COVID-19 pandemic years 2020-2021, where hospital closure for non-COVID-19 patients prevented access to perform these blood analyses. Of those missing CD4 cell count at ART initiation, most (*n =*137, 61%) belonged to the initial calendar period 2005-2009. Thirty-one (3·8%) of the excluded individuals died after ART initiation with a high proportion having a history of IDU (58%). This population differs from our overall study population being probably more prone to non-adherence to care and ART. Overall, these differences in the excluded population represent small absolute numbers and it is unlikely that it would have affected our results. Third, the availability and prescribing practices of INSTI have varied over time. We added calendar time to our models to adjust for these differences over time. However, we cannot rule out bias, especially residual channelling bias. When assessing the risk of incomplete immune recovery (two-year CD4 cell count ≤ 500 cells/μL) in LP we found 30% lower odds when initiating ART with an INSTI-based regimen. This trend was maintained when we also adjusted for calendar time, however, the results did not reach statistical significance, possibly due to lack of power. Other residual confounders such as viral resistance, lifestyle factors, or comorbidities other than the ones included were not examined, though these factors would be unlikely to have a significant impact on the results. Forth, the size of the study cohort is little with regard to a rare event like death, so risk of type II errors cannot be excluded. Finally, the lack of access to cause of death information is a limitation for the detailed interpretation of the mortality figures.

In conclusion, two-year CD4 count was inversely correlated with the mortality risk, and LP with a two-year CD4 cell count above 500 cells/µL, irrespective of their initial CD4 cell count, had the same long-term survival as non-LP. Immune recovery at this time point could be used as an early prediction of long-term survival. Despite being diagnosed at a late stage with severe immune damage, LP will regain the possibility of achieving a similar prognosis as non-LP if there is an early optimal immune recovery. In our cohort of LP, INSTI-based regimens were associated with a significantly improved two-year immune recovery and lower long-term mortality, thus being a modifiable factor to improve immune response and survival in these patients. Our data support positioning INSTI-based ART as preferred regimens, particularly in late HIV presenters, though further studies are needed to confirm these findings. Whether our results can be extrapolated to dolutegravir-based two-drug regimens will also need to be explored further.

Finally, our findings highlight the need for intensified public health efforts to improve timely HIV diagnosis and the need to continue with studies to identify modifiable determinants for immune recovery to improve survival in LP.

## Contributors

J Aceitón, Y Díaz, S Moreno-Fornés had access to the raw data and performed the initial data management. R Martin-Iguacel conducted the research and analysed the data. R Martin-Iguacel wrote the first draft of the manuscript under the supervision of JM Llibre. All authors contributed to the interpretation of the results and revision of the manuscript and gave their final approval to the manuscript.

## Data sharing statement

The data collected for this study are available from the Centre for Epidemiological Studies of Sexually Transmitted Diseases and HIV/AIDS in Catalonia (CEEISCAT), the coordinating centre of the PISCIS cohort study and from each of the collaborating hospitals upon request. Requests can be made via https://pisciscohort.org/contacte/.

The study protocol, the statistical codebook and dofiles for the analysis can be requested from RMI (raquel@bisaurin.org).

## Declaration of interests

All authors have completed the ICMJE uniform disclosure form at www.icmje.org/coi_disclosure.pdf and declare: no support from any organization for the submitted work; no financial relationships with any organizations that might have an interest in the submitted work in the previous three years; no other relationships or activities that could appear to have influenced the submitted work.

RMI has received consulting honoraria and/or research grants from GSK, MSD, and Lundbeck, outside the submitted work. RMI has received payment for travel and congress assistance from MSD, outside the present work.

JMM has received consulting honoraria and/or research grants from AbbVie, Angelini, Contrafect, Cubist, Genentech, Gilead Sciences, Jansen, Lysovant, Medtronic, MSD, Novartis, Pfizer, and ViiV Healthcare, outside the submitted work.

JML has received payments or honoraria for lectures, presentations or speaker's bureaus from Janssen-Cilag, Gilead Sciences, and ViiV Healthcare, outside of the present work.

## Appendix Supplementary materials

Image, application 1
