# Supplementary material for: Determinants of long-term survival in late HIV presenters: The prospective PISCIS cohort study
Source: eClinicalMedicine. 2022 Aug 3;52:101600. doi: 10.1016/j.eclinm.2022.101600 (PMC9358427; doi:10.1016/j.eclinm.2022.101600)
Supplement: Supplementary file 1 [file mmc1.docx]

**Supplementary material**

**Testing model assumptions**

**Poisson regression assumptions:**

- Response variable is count data (0 or 1).

- Explanatory variables are continuous, dichotomous, or ordinal.

- Observations are independent

- Event rate is constant within each interval of follow-up

-Counts follow a Poisson distribution. We check for equidispersion: the mean value of data equals to the variance value (mean= 0·0415594, variance= 0·0398469).

We also check for overdispersion using a negative binomial model. The dispersion parameter *alpha* was obtained from a negative binomial model. The *alpha* value was 0·032799 (95%CI: 0·0303581-0·0354362). The likelihood-ratio chi-square test that the dispersion parameter alpha is equal to zero was p<0·0001, thus suggesting that the response variable is not over-dispersed and is sufficiently described by the simpler Poisson distribution.

We performed both Poisson and exponential-Poisson regression. When testing the model fitness, the exponential-Poisson regression, with parametric survival-time model (STATA command *streg*), had a better model fitness than a simple Poisson model (AIC= 841 in exponential- Poisson regression vs AIC=968 Poisson regression). For this reason, we chose to use exponential-Poisson regression.

**Testing for collinearity**

We found no evidence of collinearity between viral load at two years (>200 c/ml versus ≤200 c/ml) and 2-years CD4 cell count (LP with CD4 cell count <200 cells/μL, 200-500 cells/μL, and >500 cells/μL and non-LP with CD4 cell count ≤500 cells/μL, and >500 cells/μL) (STATA command: *correlate;* estimate: -0·1).

**Propensity score matching**

We performed a propensity score matching one-to-one nearest-neighbor matching with caliper 0·2 on each sample with the covariates described in methods.

We used the following command in STATA:

*psmatch2 INSTI covariates, out (death) common caliper(0·2) noreplace*

*pstest covariates, both graph*

**Table S1A. Modified Charlson comorbidity index used in the study.**

| Comorbid conditions | Score | Number of patients with  comorbid condition  at 2 years after ART initiation  N=2719 (%) | Non-Late presenters  N (%) | Late presenters  N (%) | Number of deaths N (%) |
| --- | --- | --- | --- | --- | --- |
| Myocardial infarction | 1 | 25 (0·9) | 11 (0·9) | 14 (1·0) | 5 (20·0) |
| Congestive heart failure | 1 | 26 (1·0) | 13 (1·0) | 13 (0·9) | 5 (19·2) |
| Peripheral vascular disease | 1 | 8 (0·3) | 4 (0·31) | 4 (0·28) | 3 (37·5) |
| Cerebrovascular disease | 1 | 40 (1·5) | 12 (0·9) | 28 (1·9) | 2 (5·0) |
| Dementia | 1 | 13 (0·5) | 2 (0·2) | 11 (0·8) | 4 (30·8) |
| Chronic pulmonary disease | 1 | 55 (2·0) | 24 (1·9) | 31 (2·2) | 13 (23·6) |
| Diabetes | 1 | 52 (1·9) | 22 (1·7) | 30 (2·1) | 7 (13·5) |
| Liver disease | 1 | 232 (8·5) | 79 (6·2) | 153 (10·6) | 30 (12·9) |
| Renal disease | 2 | 31 (1·1) | 16 (1·3) | 15 (1·0) | 3 (9·7) |
| Solid organ malignancy | 2 | 142 (5·2) | 47 (3·7) | 95 (6·6) | 18 (12·7) |
| Haematological malignancy | 2 | 30 (1·1) | 9 (0·7) | 21 (1·5) | 5 (16·7) |

**Table S1B. Comorbidity characteristics at ART initiation and at 2 years after ART initiation.**

| Study population according to initial CD4 cell count | | | | Study population according to immune recovery 2 years after ART initiation | | | | |
| --- | --- | --- | --- | --- | --- | --- | --- | --- |
|  | **Non-late presenters**  **N=1278**  **(47·0%)** | **Late presenters**  **N=1441**  **(53·0%)** | **p-value** | **Late presenters**  **N=1441** | | | | **Non-late presenters** |
|  |  |  |  | **CD4 cell count (cells/µ)** | | | | **CD4 cell count (cells/µ)** |
|  |  |  |  | **>500**  **n=637 (44·2%)** | **>350-500**  **n=374 (26·0%)** | **≥200-350 n=304**  **(21·1%)** | **<200**  **n=126 (8·7%)** | **>500 cells/µL**  **N=1145** |
| Comorbidity at ART initiation, n (%) |  |  |  |  |  |  |  |  |
| Cardiovascular disease | 21 (1·6) | 31 (2·2) | 0·33 | 5 (0·8) | 8 (2·1) | 13 (4·3) | 5 (4·0) | 18 (1·6) |
| Chronic kidney disease | 13 (1·0) | 8 (0·6) | 0·17 | 2 (0·3) | 3 (0·8) | 1 (0·3) | 2 (1·6) | 13 (1·1) |
| Chronic liver disease | 54 (4·2) | 92 (6·4) | 0·013 | 23 (3·6) | 24 (6·4) | 25 (8·2) | 20 (15·9) | 45 (3·9) |
| COPD, emphysema, chronic bronchitis | 16 (1·3) | 21 (1·5) | 0·65 | 6 (0·9) | 3 (0·8) | 9 (3·0) | 3 (2·4) | 14 (1·2) |
| Diabetes | 17 (1·3) | 18 (1·3) | 0·85 | 6 (0·9) | 7 (1·9) | 3 (1·0) | 2 (1·6) | 14 (1·2) |
| Hematological neoplasm | 5 (0·4) | 8 (0·6) | 0·54 | 2 (0·3) | 3 (0·8) | 2 (0·7) | 1 (0·8) | 4 (0·4) |
| Solid neoplasm | 21 (1·6) | 45 (3·1) | 0·012 | 18 (2·8) | 11 (2·9) | 9 (3·0) | 7 (5·6) | 18 (1·6) |
| Dementia | 2 (0·2) | 5 (0·4) | 0·33 | 0 | 0 | 5 (1·6) | 0 | 2 (0·2) |
| Comorbidity at 2 years after ART initiation, n (%) |  |  |  |  |  |  |  |  |
| Cardiovascular disease | 39 (3·1) | 55 (3·8) | 0·28 | 10 (1·6) | 13 (3·5) | 18 (5·9) | 14 (11·1) | 31 (2·7) |
| Chronic kidney disease | 16 (1·3) | 15 (1·0) | 0·61 | 3 (0·5) | 6 (1·6) | 2 (0·7) | 4 (3·2) | 16 (1·4) |
| Chronic liver disease | 79 (6·2) | 153 (10·6) | <·0001 | 43 (6·8) | 38 (10·2) | 41 (13·5) | 31 (24·6) | 64 (5·6) |
| COPD, emphysema, chronic bronchitis | 24 (1·9) | 31 (2·2) | 0·61 | 11 (1·7) | 5 (1·3) | 10 (3·3) | 5 (4·0) | 21 (1·8) |
| Diabetes | 22 (1·7) | 30 (2·1) | 0·49 | 10 (1·6) | 10 (2·7) | 7 (2·3) | 3 (2·4) | 19 (1·7) |
| Hematological neoplasm | 9 (0·7) | 21 (1·5) | 0·061 | 5 (0·8) | 6 (1·6) | 5 (1·6) | 5 (4·0) | 8 (0·7) |
| Solid neoplasm | 47 (3·7) | 95 (6·6) | 0·001 | 26 (4·1) | 19 (5·1) | 31 (10·2) | 19 (15·1) | 42 (3·7) |
| Dementia | 2 (0·2) | 11 (0·8) | 0·022 | 2 (0·3) | 1 (0·3) | 5 (1·6) | 3 (2·4) | 2 (0·2) |
| Modified Charlson comorbidity  index at ART initiation |  |  |  |  |  |  |  |  |
| 0 | 1158 (90·6) | 1262 (87·6) | 0·012 | 583 (91·5) | 328 (87·7) | 256 (84·2) | 95 (75·4) | 1041 (90·9) |
| 1 | 76 (6·0) | 108 (7·5) | 0·11 | 32 (5·0) | 25 (6·7) | 31 (10·2) | 20 (15·9) | 67 (5·9) |
| 2-3 | 36 (2·8) | 60 (4·2) | 0·058 | 20 (3·1) | 18 (4·8) | 14 (4·6) | 8 (6·4) | 30 (2·6) |
| ≥4 | 8 (0·6) | 11 (0·8) | 0·67 | 2 (0·3) | 3 (0·8) | 3 (1·0) | 3 (2·4) | 7 (0·6) |
| Modified Charlson comorbidity index at 2 years after ART initiation |  |  |  |  |  |  |  |  |
| 0 | 1094 (85·6) | 1146 (79·5) | 0·012 | 551 (86·5) | 301 (80·5) | 221 (72·7) | 73 (57·9) | 988 (86·3) |
| 1 | 107 (8·4) | 158 (11·0) | 0·11 | 52 (8·2) | 39 (10·4) | 42 (13·8) | 25 (19·8) | 90 (7·9) |
| 2-3 | 62 (4·9) | 106 (7·4) | 0·058 | 29 (4·6) | 26 (7·0) | 32 (10·5) | 19 (15·1) | 53 (4·6) |
| ≥4 | 15 (1·2) | 31 (2·2) | 0·67 | 5 (0·8) | 8 (2·1) | 9 (3·0) | 9 (7·1) | 14 (1·2) |

**Table S2A. Comparison between the individuals included in the study who died and those excluded because they died before study inclusion (2 years after ART initiation) or after study inclusion but did not have CD4 cell count available at ART initiation and/or at 2 years after.**

|  | Individuals included in the study who died during the study period  N=113 | Individuals excluded from the  study: died before study inclusion (within 2 years from ART initiation)  N=70 | Individuals excluded from  the study: died after  study inclusion but had no  available CD4 count  after ART initiation  N=31 |
| --- | --- | --- | --- |
| CD4 cell count at HIV diagnosis, median (IQR) | 186 (80-301) | 136 (50-277) | 239 (148-482) |
| Male gender, n (%) | 99 (87·6) | 55 (78·6) | 28 (90·3) |
| Age at ART initiation, median (IQR) | 48 (39-55) | 44 (38-51) | 44 (38-47) |
| Region of birth, n (%) |  |  |  |
| Spain | 89 (80·2) | 55 (82·1) | 27 (87·1) |
| Europe | 10 (9·0) | 4 (6·0) | 1 (3·2) |
| Africa | 4 (3·6) | 1 (1·5) | 2 (6·5) |
| America | 8 /7·2) | 7 (10·5) | 1 (3·2) |
| Asia | 0 | 0 | 0 |
| HIV risk group, n (%) |  |  |  |
| MSM | 30 (26·6) | 22 (31·4) | 5 (16·1) |
| Heterosexual men | 37 (32·7) | 12 (17·4) | 7 (22·6) |
| Women | 10 (8·9) | 8 (11·4) | 1 (3·2) |
| IDU | 25 (22·1) | 19 (27·1) | 18 (58·1) |
| Other | 11 (9·7) | 9 (12·9) | 0 |
| Calendar period of death, n (%) |  |  |  |
| 2005-2009 | 6 (5·3) | 35 (50·0) | 3 (9·7) |
| 2010-2014 | 28 (24·8) | 16 (22·9) | 8 (25·8) |
| 2015-2021 | 79 /70·0) | 19 (27·1) | 20 (64·5) |
| Individuals with AIDS-defining event at HIV diagnosis, n (%) | 26 (23·0) | 19 (27·1) | 6 (19·4) |
| Modified Charlson comorbidity index at ART initiation, n (%) |  |  |  |
| 0 | 78 (69·0) | 44 (62·9) | 24 (77·4) |
| 1 | 19 (16·8) | 11 (15·7) | 6 (19·4) |
| 2-3 | 9 (8·0) | 11 (15·7) | 1 (3·2) |
| ≥4 | 7 (6·2) | 4 (5·7) | 0 |

AIDS, Acquired Immune Deficiency Syndrome; ART, antiretroviral therapy; IQR, interquartile range (percentiles 25^th^ and 75^th^); MSM, men who have sex with men; IDU, injection drug use.

**Table S2B. Distribution over calendar time of individuals excluded from the analysis because of lack of availability of CD4 cell count at ART initiation and/or 2 years after (n=817).**

|  | Individuals with missing CD4 cell count at ART initiation, n (%)  N=223 |  | Individuals with missing CD4 cell count 2 years after ART initiation, n (%)  N=708 |
| --- | --- | --- | --- |
| Calendar time |  | Calendar time |  |
| 2005-2009 | 137 (61·4) | 2005-2009 | 102 (14.9) |
| 2010-2014 | 56 (25·1) | 2010-2014 | 190 (27.8)) |
| 2015-2019 | 30 (13·5) | 2015-2019 | 110 (16.11) |
|  |  | 2020-2021 | 281 (41.1) |

**Table S3. Mortality rate depending on the immune recovery 2 years after ART initiation in late presenters compared to non-late presenters with CD4 cell count >500 cells/µL 2 years after ART initiation.**

|  | All individuals  (n=2719) | | | | | Late presenters (n=1441) | Non-late presenters (n=1278) |
| --- | --- | --- | --- | --- | --- | --- | --- |
|  | Deaths | PYR | MR per 1000 PYR (95% CI) | MRR (95% CI) | aMRR (95% CI) | aMRR (95% CI) | aMRR (95% CI) |
| Total | 113 | 16593·1 | 6·8 (5·7-8·2) |  |  |  |  |
| Gender |  |  |  |  |  |  |  |
| Female | 14 | 2591·4 | 5·4 (3·2-9·1) | Ref (1) |  |  |  |
| Male | 99 | 14001·7 | 7·1 (5·8-8·6) | 1·31 (0·75-2·29) |  |  |  |
| Age at baseline |  |  |  | 1·08 (1·07-1·10) | 1·07 (1·05-1·09) | 1·07 (1·04-1·09) | 1·08 (1·03-1·13) |
| Country of birth |  |  |  |  |  |  |  |
| Spain | 89 | 8838·6 | 10·1 (8·2-12·4) | Ref (1) |  |  |  |
| Europe | 10 | 1151·9 | 8·7 (4·7-16·1) | 0·86 (0·45-1·66) |  |  |  |
| Africa | 4 | 741·7 | 5·4 (2·0-14·4) | 0·54 (0·20-1·46) |  |  |  |
| America | 8 | 3348·8 | 2·4 (1·2-4·8) | 0·24 (0·12-0·49) |  |  |  |
| Asia | 0 | 152·2 | 0 | - |  |  |  |
| HIV risk group |  |  |  |  |  |  |  |
| MSM | 30 | 9738·9 | 3·1 (2·2-4·4) | Ref (1) | Ref (1) | Ref (1) | Ref (1) |
| Heterosexual men | 37 | 2533·4 | 14·6 (10·6-20·2) | 4·74 (2·93-7·67) | 1·94 (1·13-3·34) | 1·61 (0·87-2·98) | 4·78 (1·68-13·62) |
| Women | 10 | 2108·0 | 4·7 (2·6-8·8) | 1·54 (0·75-3·15) | 0·90 (0·42-1·93) | 0·99 (0·43-2·27) | 0·39 (0·04-3·60) |
| IDU | 25 | 1256·9 | 19·9 (13·4-29·4) | 6·46 (3·80-10·98) | 2·74 (1·49-5·03) | 2·15 (1·07-4·30) | 4·70 (1·26-17·48) |
| Unknown/other | 11 | 955·9 | 11·5 (6·4-20·8) | 3·74 (1·87-7·45) | 1·51 (0·69-3·33) | 1·59 (0·69-3·66) | - |
| Two-year CD4 cell count after ART initiation (cells/ µL) |  |  |  |  |  |  |  |
| LP, CD4 <200 | 29 | 802·4 | 36·1 (25·1-52·0) | 12·66 (6·96-23·03) | 4·59 (2·25-9·37) | 4·67 (2·29-9·50) | - |
| LP, CD4 200-500 | 47 | 4776·2 | 9·8 (7·4-13·1) | 3·45 (1·98-6·00) | 1·95 (1·06-3·61) | 1·93 (1·05-3·57) | - |
| LP, CD4>500 | 14 | 4236·5 | 3·3 (2·0-5·6) | 1·16 (0·57-2·35) | 1·05 (0·50-2·21) | Ref (1) | - |
| Non-LP, CD4 <500 | 6 | 824·8 | 7·3 (3·3-16·2) | 2·55 (1·00-6·46) | 1·97 /0·75-5·21) |  | 1·89 (0·63-5·63) |
| Non-LP, CD4 >500 | 17 | 5953·3 | 2·9 (1·8-4·6) | Ref (1) | Ref (1) | - | Ref (1) |
| HIV viral load >200 c/ml 2 years after ART initiation |  |  |  |  |  |  |  |
| Yes | 15 | 1100·5 | 13·6 (8·2-22·6) | 2·28 (1·32-3·94) | 1·78 (0·99-3·19) | 1·82 (0·96-3·45) | 1·96 (0·41-9·28) |
| No | 89 | 14893·3 | 6·0 (4·9-7·4) | Ref (1) | Ref (1) | Ref (1) | Ref (1) |
| INSTI as ART-regimen initiated in the first 2 years |  |  |  |  |  |  |  |
| Yes | 21 | 3851·0 | 5·5 (3·6-8·4) | 0·76 (0·47-1·21) | 0·54 (0·31-0·93) | 0·61 (0·33-1·13) | 0·25 (0·06-1·11) |
| No | 92 | 12742·1 | 7·2 (5·9-8·9) | Ref (1) | Ref (1) | Ref (1) | Ref (1) |
| Modified Charlson comorbidity index at 2 years after ART initiation |  |  |  |  |  |  |  |
| 0 | 63 | 13916·9 | 4·5 (3·5-5·8) | Ref (1) | Ref (1) | Ref (1) | Ref (1) |
| 1 | 20 | 1519·2 | 13·2 (8·5-20·4) | 2·91 (1·76-4·81) | 1·40 (0·80-2·45) | 1·58 (0·86-2·91) | 0·94 (0·19-4·65) |
| 2-3 | 17 | 963·8 | 17·6 (11·0-28·4) | 3·90 (2·28-6·66) | 1·57 (0·86-2·86) | 1·67 (0·87-3·21) | 0·82 (0·15-4·48) |
| ≥ 4 | 13 | 193·2 | 67·3 (39·1-115·9) | 14·86 (8·18-27·0) | 3·96 (1·84-8·50) | 3·84 (1·60-9·17) | 3·53 (0·53-23·63) |
| Calendar time |  |  |  |  |  |  |  |
| 2005-2009 | 6 | 428·5 | 14·0 (6·3-31·2) | 2·13 (0·93-4·89) |  |  |  |
| 2010-2014 | 28 | 4137·1 | 6·8 (4·7-9·8) | 1·03 (0·67-1·59) |  |  |  |
| 2015-2021 | 79 | 12027·5 | 6·6 (5·3-8·2) | Ref (1) |  |  |  |
| AIDS defining event in the first 2 years after ART initiation |  |  |  |  |  |  |  |
| Yes | 28 | 1660·1 | 16·9 (11·6-24·4) | 2·96 (1·93-4·54) | 1·36 (0·86-2·15) | 1·23 (0·74-2·03) | 2·54 (0·70-9·21) |
| No | 85 | 14933·0 | 5·7 (4·6-7·0) | Ref (1) | Ref (1) | Ref (1) | Ref (1) |
| Income |  |  |  |  |  |  |  |
| No economic deprivation | 42 | 8195·8 | 5·1 (3·8-6·9) | Ref (1) |  |  |  |
| Mild economic deprivation | 26 | 3276·8 | 8·0 (5·4-11·7) | 1·55 (0·95-2·53) |  |  |  |
| Moderate/severe economic deprivation | 42 | 4629·6 | 9·1 (6·7-12·3) | 1·77 (1·15-2·72) |  |  |  |

AIDS, Acquired Immune Deficiency Syndrome; aMRR, adjusted mortality rate ratio; ART, antiretroviral therapy; INSTI, integrase strand transfer inhibitor; MSM, men who have sex with men; IDU, injection drug use; MR, mortality rate; MRR, mortality rate ratio; LP, late presenters.

^1^ CD4 200-350 cells/µL in non-late presenters

^2^ CD4 >350-500 cells/µL in non-late presenters

**Figure S1. Kaplan-Meier curves for overall survival by immune recovery two years after ART initiation excluding patients with viral load >200 c/ml or with missing values at 2 years after ART initiation (n= 349)(non-late presenter with CD4 cell count >500 cells/µL versus late presenters with CD4 cell count >500 cells/µL, late presenters with CD4 cell count >350-500 cells/µL, late presenters with CD4 cell count 200-350 cells/µL and late presenters with CD4 cell count <200 cells/µL at 2 years after ART initiation).**

**Table S4. Mortality rates upon immune recovery 2 years after initiation of antiretroviral therapy stratified by CD4 cell count at antiretroviral therapy (ART) initiation.**

| **Two-year CD4 cell count after**  **ART initiation** | **CD4 cell count at ART initiation** | **N (%)** | **Deaths** | **Mortality rate (MR)**  **(95% CI)** | **MRR**  **(95% CI)** | **aMRR (95% CI)** |
| --- | --- | --- | --- | --- | --- | --- |
| CD4 cell count >500 cells/µL, N=1782 |  |  |  |  |  |  |
|  | CD4 cell count < 100 cells/µL (n=383) | 67 (17·5) | 3 | 6·3 (2·0-19·5) | 2·20 (0·65-7·51) | 1·15 (0·30-4·35) |
|  | CD4 cell count 100-199 cells/µL (n=314) | 109 (34·7) | 3 | 4·3 (1·4-13·4) | 1·51 (0·44-5·16) | 1·18 (0·34-4·16) |
|  | CD4 cell count 200-350 cells/µL (n=744) | 461 (62·0) | 8 | 2·6 (1·3-5·2) | 0·91 (0·39-2·12) | 1·08 (0·44-2·60) |
|  | CD4 cell count >350 cells/µL (n=1278) | 1145 (89·6) | 17 | 2·9 (1·8-4·6) | Ref (1) | Ref (1) |
| CD4 cell count >350-500 cells/µL, N=478 |  |  |  |  |  |  |
|  | CD4 cell count < 100 cells/µL (n=383) | 92 (24·0) | 5 | 8·4 (3·5-20·3) | 1·10 (0·32-3·80) | 0·61 (0·16-2·31) |
|  | CD4 cell count 100-199 cells/µL (n=314) | 97 (30·9) | 8 | 12·4 (6·2-24·8) | 1·62 (0·53-4·94) | 1·27 (0·38-4·22) |
|  | CD4 cell count 200-350 cells/µL (n=744) | 185 (24·9) | 12 | 9·2 (5·2-16·2) | 1·20 (0·42-3·40) | 0·87 (0·29-2·60) |
|  | CD4 cell count >350 cells/µL (n=1278) | 104 (8·1) | 5 | 7·7 (3·2-18·4) | Ref (1) | Ref (1) |
| CD4 cell count 200-350 cells/µL, N=328 |  |  |  |  |  |  |
|  | CD4 cell count < 100 cells/µL (n=383) | 133 (34·7) | 9 | 9·2 (4·8-17·7) | 1·36 (0·17-10·73) | 0·91 (0·11-7·73) |
|  | CD4 cell count 100-199 cells/µL (n=314) | 88 (28·0) | 7 | 10·6 (5·1-22·3) | 1·56 (0·19-12·71) | 0·93 (0·10-8·35) |
|  | CD4 cell count 200-350 cells/µL (n=744) | 83 (11·2) | 6 | 10·0 (4·5-22·3) | 1·47 (0·18-12·25) | 0·80 (0·09-7·39) |
|  | CD4 cell count >350 cells/µL (n=1278) | 24 (1·9) | 1 | 6·8 (1·0-48·2) | Ref (1) | Ref (1) |
| CD4 cell count <200 cells/µL, N=131 |  |  |  |  |  |  |
|  | CD4 cell count < 100 cells/µL (n=383) | 91 (23·8) | 20 | 33·7 (21·7-52·2) | 1·36 (0·32-5·83) | 2·20 (0·25-19·12) |
|  | CD4 cell count 100-199 cells/µL (n=314) | 20 (6·4) | 7 | 54·7 (26·1-114·7) | 2·21 (0·46-10·65) | 2·12 (0·21-20·90) |
|  | CD4 cell count 200-350 cells/µL (n=744) | 15 (2·0) | 2 | 24·7 (6·2-98·8) | Ref (1) | Ref (1) |
|  | CD4 cell count >350 cells/µL (n=1278) | 5 (0·4) | 0 | - | - | - |

aMMR, adjused mortality rates ratios.

aMRR are adjusted for age (time-updated), HIV risk group, calendar time (time-updated), modified charlson comorbidity index at 2 years after ART initiation, and HIV viral load > or ≤ 200 c/ml at 2 years after ART initiation.

**Table S5. Crude and adjusted mortality according to exposure to INSTI-based regimens in treatment naive individuals stratified by 1) late and non-late presenters surviving the first 2 years, 2) all individuals surviving the first 2 years and performing propensity score matching and 3) all individuals initiating ART and performing propensity score weighting.**

^
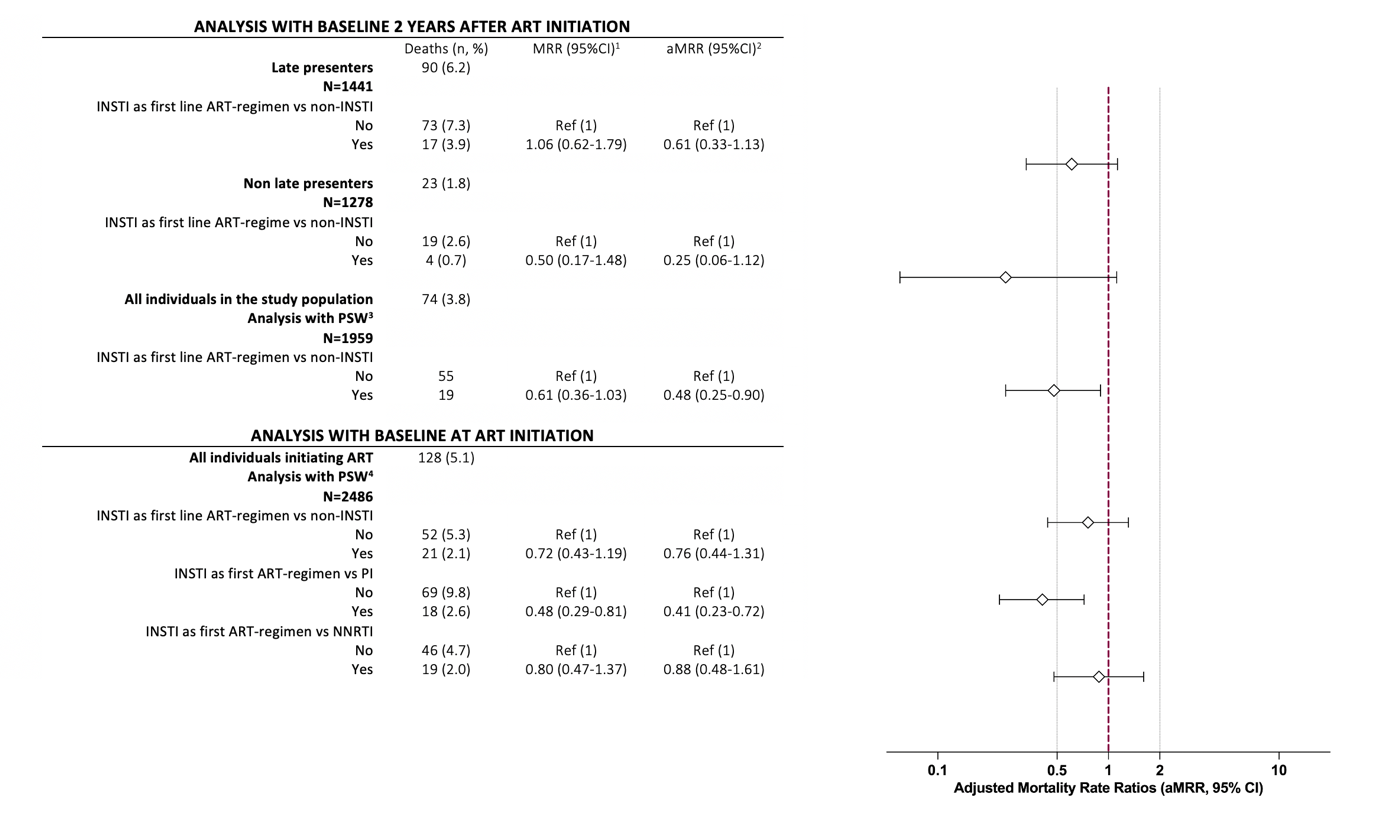
^

ART, antiretroviral therapy; INSTI, integrase strand transfer inhibitor; NNRTI, non-nucleoside reverse transcriptase inhibitors; PI, protease inhibitor.

^1^ MRR, mortality rate ratio.

^2^ aMRR, adjusted mortality rate ratio. The multivariate analysis was adjusted for age at baseline (2 years after ART initiation), HIV risk group, CD4 cell count recovery at 2 years after ART initiation, HIV viral load > 200 c/ml vs ≤200 c/ml at 2 years, INSTI-based regimen as first-line regimen in the first 2 years, modified Charlson comorbidity index at baseline, history of AIDS-defining event at baseline and calendar time (time-updated).

In the first part of the table, mortality analyses include only late presenters (n=1441) or non-late presenters (n=1278).

^3^Sensitivity analysis including all individuals in the study period (n=2719). We performed propensity score matching (PSM) on gender, age, CD4 cell count at ART initiation, modified Charlson comorbidity index at ART initiation, and AIDS-defining event at ART initiation (n=1959, deaths 74 (3·8%)). Subsequently, we performed Poisson exponential regression adjusting for age, HIV risk group, CD4 cell count recovery at 2 years after ART initiation, HIV viral load > 200 c/ml vs ≤200 c/ml at 2 years, INSTI-based regimen initiated in the first 2 years, modified Charlson comorbidity index at baseline, history of AIDS-defining event at baseline, and calendar time (time-updated).

^4^ Sensitivity analysis including all individuals who started ART in the study period (N=3379 and 197 deaths (5·8%). We performed propensity score matching (PSM) on gender, age, CD4 cell count at ART initiation, modified Charlson comorbidity index at ART initiation, and AIDS-defining event at ART initiation (n=2486, 128 deaths (5·1%)). Subsequently, we performed Poisson exponential regression adjusting for age, HIV risk group, CD4 cell count at ART initiation, INSTI-based regimen as first-line regimen, modified Charlson comorbidity index at ART initiation, AIDS-defining event at ART initiation, and calendar time (time-updated).

**Table S6. Mortality in non-late presenters upon immune recovery 2 years after ART initiation**

1. **non-late presenters with CD4 < 700 cells/µL 2 years after ART initiation (n=423) vs non-late presenters with CD4 ≥ 700 cells/µL 2 years after ART initiation (n=855).**

|  | Deaths | PYR | MR per 1000 PYR (95% CI) | MRR (95% CI) | aMRR (95% CI) |
| --- | --- | --- | --- | --- | --- |
| Total | 23 | 6778·1 | 3·4 (2·3-5·1) |  |  |
| Age (time-updated) |  |  |  |  |  |
| <50 years | 11 | 5458·7 | 2·0 (1·1-3·6) | Ref (1) | Ref (1) |
| 50-59 years | 7 | 977·8 | 7·2 (3·4-15·0) | 3·55 (1·38-9·16) | 2·52 (0·96-6·59) |
| ≥ 60 years | 5 | 341·6 | 14·6 (6·1-35·2) | 7·26 (2·52-20·90) | 5·66 (1·93-16·57) |
| HIV risk group |  |  |  |  |  |
| MSM | 8 | 4783·5 | 1·7 (0·8-3·3) | Ref (1) | Ref (1) |
| Heterosexual men | 8 | 655·1 | 12·2 (6·1-24·4) | 7·30 (2·74-19·46) | 5·72 (2·11-15·44) |
| Women | 2 | 722·8 | 2·8 (0·7-11·1) | 1·65 (0·35-7·79) | 1·42 (0·30-6·71) |
| IDU | 5 | 332·6 | 15·0 (6·3-36·1) | 8·99 (2·94-27·48) | 7·08 (2·28-21·95) |
| Unknown/other | 0 | 284·0 | - | - | - |
| CD4 cell count 2 years after ART initiation (cells/µL) |  |  |  |  |  |
| Non-late presenters, CD4 <700 cells/µL | 14 | 2503·8 | 5·6 (3·3-9·4) | Ref (1) | Ref (1) |
| Non-late presenters, CD4 ≥700 cells/µL | 9 | 4274·3 | 2·1 (1·2-4·4) | 0·38 (0·16-0·87) | 0·49 (0·21-1·16) |

1. **non-late presenters with CD4 ≥500 and <700 cells/µL 2 years after ART initiation (n=290) vs non-late presenters with CD4 ≥ 700 cells/µL 2 years after ART initiation (n=855).**

|  | Deaths | PYR | MR per 1000 PYR (95% CI) | MRR (95% CI) | aMRR (95% CI) |
| --- | --- | --- | --- | --- | --- |
| Total | 17 | 5953·3 | 2·9 ((1·8-4·6) |  |  |
| Age (time-updated) |  |  |  |  |  |
| <50 years | 8 | 4841·7 | 1·6 (0·8-3·3) | Ref (1) | Ref (1) |
| 50-59 years | 5 | 842·8 | 5·9 (2·5-14·3) | 3·59 (1·17-10·97) | 2·49 (0·80-7·77) |
| ≥ 60 years | 4 | 268·8 | 14·9 (5·6-39·7) | 9·01 (2·71-29·91) | 6·42 (1·85-22·33) |
| HIV risk group |  |  |  |  |  |
| MSM | 6 | 4384·1 | 1·4 (0·6-3·0) | Ref (1) | Ref (1) |
| Heterosexual men | 7 | 556·6 | 12·6 (6·0-26·4) | 9·19 (3·09-27·34) | 6·80 (2·22-20·81) |
| Women | 2 | 548·4 | 3·6 (0·9-14·6) | 2·66 (0·54-13·20) | 2·14 (0·42-10·79) |
| IDU | 2 | 246·5 | 8·1 (2·0-32·4) | 5·92 (1·20-29·37) | 5·70 (1·13-28·76) |
| Unknown/other | 0 | 217·8 | - | - | - |
| CD4 cell count 2 years after ART initiation (cells/µL) |  |  |  |  |  |
| Non-late presenters, CD4 ≥500 and <700 cells/µL | 8 | 1679·0 | 4·8 (2·4-9·5) | Ref (1) | Ref (1) |
| Non-late presenters, CD4 ≥700 cells/µL | 9 | 4274·3 | 2·1 (1·1-4·0) | 0·44 (0·17-1·15) | 0·55 (0·21-1·47) |

aMRR, adjusted mortality rate ratio; ART, antiretroviral therapy; MSM, men who have sex with men; IDU, injection drug use; MR, mortality rate; MRR, mortality rate ratio; PYR, person-years at risk.

**Table S7. Mortality rate depending on the immune recovery 2 years after ART initiation in late presenters compared to non-late presenters with two-year CD4 cell count ≥700 cells/µL.**

|  | Deaths | MR per 1000 PYR (95% CI) | MRR (95% CI) | aMRR (95% CI) |
| --- | --- | --- | --- | --- |
| Total | 113 | 6·8 (5·7-8·2) |  |  |
| Age (time-updated) |  |  |  |  |
| <50 years | 47 | 3·8 (2·9-5·1) | Ref (1) | Ref (1) |
| 50-59 years | 30 | 9·4 (6·6-13·5) | 2·45 (1·55-3·88) | 1·47 (0·86-2·49) |
| ≥ 60 years | 36 | 30·4 (21·9-42·1) | 7·90 (5·12-12·20) | 5·10 (3·01-8·67) |
| HIV risk group |  |  |  |  |
| MSM | 30 | 3·1 (2·2-4·4) | Ref (1) | Ref (1) |
| Heterosexual men | 37 | 14·6 (10·6-20·2) | 4·74 (2·93-7·67) | 2·09 (1·16-3·76) |
| Women | 10 | 4·7 (2·6-8·8) | 1·54 (0·75-3·15) | 1·11 (0·51-2·45) |
| IDU | 25 | 19·9 (13·4-29·4) | 6·46 (3·80-10·98) | 2·91 (1·48-5·73) |
| Unknown/other | 11 | 11·5 (6·4-20·8) | 3·74 (1·87-7·45) | 1·75 (0·77-3·98) |
| CD4 cell count 2 years after ART initiation (cells/µL) |  |  |  |  |
| LP, CD4 <200 | 29 | 36·1 (26·1-52·0) | 17·16 (8·13-36·26) | 6·55 (2·67-16·06) |
| LP, CD4 200-500 | 47 | 9·8 (7·4-14·1) | 4·67 (2·29-9·54) | 2·70 (1·18-6·20) |
| LP, CD4>500-699 | 10 | 3·8 (2·1-7·1) | 1·82 (0·74-4·48) | 1·56 (0·59-4·15) |
| LP, CD4 ≥700 | 4 | 2·5 (0·9-6·5) | 1·17 (0·36-3·79) | 1·15 (0·34-3·98) |
| Non-LP, CD4 ≥700 | 9 | 2·1 (1·1-4·0) | Ref (1) | Ref (1) |
| HIV viral load >200 c/ml 2 years after ART initiation |  |  |  |  |
| Yes | 15 | 13·6 (8·2-22·6) | 2·28 (1·32-3·94) | 1·81 (0·96-3·40) |
| No | 89 | 6·0 (4·9-7·4) | Ref (1) |  |
| Modified Charlson comorbidity index at ART initiation |  |  |  |  |
| 0 | 63 | 4·5 (3·5-5·8) | Ref (1) | Ref (1) |
| 1 | 20 | 13·2 (8·5-20·4) | 2·91 (1·76-4·81) | 1·49 (0·82-2·71) |
| 2-3 | 17 | 17·6 (11·0-28·4) | 3·90 (2·28-6·66) | 2·08 (1·12-3·85) |
| ≥ 4 | 13 | 67·3 (39·1-115·9) | 14·86 (8·18-27·0) | 5·03 (2·14-11·80) |
| INSTI as ART-regimen initiated in the first 2 years |  |  |  |  |
| Yes | 21 | 5·5 (3·6-8·4) | 0·76 (0·47-1·21) | 0·62 (0·34-1·12) |
| No | 92 | 7·6 (6·2-9·3) | Ref (1) | Ref (1) |

aMRR, adjusted mortality rate ratio; ART, antiretroviral therapy; INSTI, integrase strand transfer inhibitor; MSM, men who have sex with men; IDU, injection drug use; MR, mortality rate; MRR, mortality rate ratio; LP, late presenters.

**Table S8. Main baseline characteristics before and after propensity score matching when comparing late presenters initiating ART with INSTI versus non-INSTI.**

| Individuals with CD4 cell count AT ART initiation ≤350 cells/µL | Before PSM  (n=1441) | | After PSM^1^  (n=870) | |
| --- | --- | --- | --- | --- |
| Mean bias | 12.3 | | 4.5 | |
|  | INSTI | No INSTI | INSTI | No INSTI |
| Incomplete immune response at two years, n (%) | 237 (54·5) | 567 (56·4) | 237 (54·5) | 260 (59·8) |
| Men, n (%) | 371 (85·3) | 831 (82·6) | 371 (85·3) | 380 (87·4) |
| CD4 cell count at ART initiation (cells/ µL), median (IQR) | 195 (76-283) | 211 (98-281) | 195 (76-283) | 209 (89-282) |
| CD4 cell count at ART initiation (cells/ µL) |  |  |  |  |
| CD4 cell count 350-200 |  |  | 212 (48·7) | 229 (52·6) |
| CD4 cell count 100-199 | 307 (70·6) | 571 (74·7) | 95 (21·8) | 82 (18·9) |
| CD4 cell count <100 | 128 (29·4) | 255 (25·4) | 128 (29·4) | 124 (28·5) |
| Age (years), median (IQR) | 39 (32-46) | 38 (31-45) | 39 (32-46) | 39 (31-46) |
| Age at ART initiation (years) |  |  |  |  |
| <30 | 78 (17·9) | 204 (20·3) | 78 (17·9) | 95 (21·8) |
| 30-39 | 153 (35·2) | 385 (38·3) | 153 (35·2) | 140 (32·2) |
| 40-49 | 136 (31·3) | 285 (28·3) | 136 (31·3) | 134 (30·8) |
| ≥50 | 68 (15·6) | 132 (13·1) | 68 (15·6) | 66 (15·6) |
| Modified Charlson comorbidity index at ART initiation |  |  |  |  |
| 0 | 361 (83·0) | 901 (89·6) | 361 (83·0) | 358 (82·3) |
| 1 | 40 (9·2) | 68 (6·8) | 40 (9·2) | 42 (9·7) |
| 2-3 | 28 (6·4) | 32 (3·2) | 28 (6·4) | 30 (6·9) |
| ≥4 | 6 (1·4) | 5 (0·5) | 6 (1·4) | 5 (1·2) |
| AIDS-defining event at ART initiation |  |  |  |  |
| No | 372 (85·5) | 879 (87·4) | 372 (85·5) | 381 (87·6) |
| Yes | 63 (14·5) | 127 (12·6) | 63 (14·5) | 54 (12·4) |
| Calendar period of ART initiation |  |  |  |  |
| 2005-2009 | 6 (1·4) | 270 (26·8) | 6 (1·4) | 120 (27·6) |
| 2010-2015 | 179 (41·2) | 676 (67·2) | 179 (41·2) | 285 (65·5) |
| 2015-2019 | 250 (57·5) | 60 (6·0) | 250 (57·5) | 30 (6·9) |

Abbreviations: ART, antiretroviral therapy; IQR, interquartile range (percentiles 25^th^ and 75^th^); PSM, propensity score matching.

^1^ Matching on age, gender, CD4 cell count at ART initiation, and modified Charlson comorbidity index at ART initiation.

**Table S9. Variables associated with incomplete immune restoration, defined as CD4 cell count <700 cells/µL, 2 years after ART initiation, in individuals with CD4 cell count <500 cells/µL at ART initiation**.

|  | Incomplete immune recovery  (n, %) | OR (95% CI) | aOR (95% CI)^1^ |
| --- | --- | --- | --- |
| **Individuals with CD4 cell count at ART initiation <500 cells/µL (n=1880)** | 965 (51·3) |  |  |
| Age at ART initiation (years) |  |  |  |
| <30 | 183 (40·3) | Ref (1) | Ref (1) |
| 30-39 | 344 (46·9) | 1·31 (1·03-1·66) | 1·13 (0·85-1·51) |
| 40-49 | 292 (61·1) | 2·32 (1·79-3·02) | 1·41 (1·02-1·94) |
| ≥50 | 146 (67·9) | 3·13 (2·22-4·41) | 1·65 (1·06-2·56) |
| CD4 cell count at ART initiation  (cells/ µL) |  |  |  |
| CD4 >350-499 | 250 (24·8) | Ref (1) | Ref (1) |
| CD4 >200-350 | 312 (71·4) | 7·57 (5·88-9·74) | 7·23 (5·58-9·37) |
| CD4 100-199 | 170 (88·5) | 23·43 (14·69-37·36) | 21·06 (13·05-33·99) |
| CD4 <100 | 233 (95·9) | 70·65 (36·92-135·18) | 63·10 (31·97-124·54) |
| INSTI as first-line ART regimen |  |  |  |
| No | 518 (55·1) | Ref (1) | Ref (1) |
| Yes | 447 (47·6) | 0·74 (0·62-0·89) | 0·74 (0·57-0·97) |
| AIDS-defining event at ART initiation |  |  |  |
| No | 850 (48·6) | Ref (1) | Ref (1) |
| Yes | 115 (87·8) | 7·60 (4·47-12·93) | 1·19 (0·62-2·31) |
| Gender |  |  |  |
| Men | 839 (50·4) | Ref (1) | Ref (1) |
| Women | 126 (58·3) | 0·73 (0·55-0·97) | 1·22 (0·84-1·78) |
| Modified Charlson comorbidity index at ART initiation |  |  |  |
| 0 | 793 (48·7) | Ref (1) | Ref (1) |
| 1 | 100 (66·7) | 2·11 (1·48-3·00) | 1·63 (1·05-2·53) |
| 2-3 | 59 (67·8) | 2·22 (1·40-3·52) | 1·25 (0·68-2·28) |
| ≥4 | 13 (86·7) | 6·84 (1·54-30·43) | 5·66 (1·04-30·72) |
| Calendar period of ART initiation |  |  |  |
| 2005-2009 | 131 (76·2) | Ref (1) | Ref (1) |
| 2010-2014 | 543 (52·3) | 0·34 (0·24-0·50) | 0·71 (0·45-1·13) |
| 2015-2019 | 291 (43·4) | 0·24 (0·16-0·35) | 0·56 (0·33-0·94) |

Abbreviations: aOR, adjusted odds ratio; ART, antiretroviral therapy; INSTI, integrase strand transfer inhibitor; OR, odds ratio.

^1^ Adjusted for age, gender, CD4 cell count at ART initiation, INSTI-based regimen as first-line regimen, AIDS-defining event, modified Charlson comorbidity index, calendar time.
